# Supplementary material for: Heat Stress Induces Alterations in Gene Expression of Actin Cytoskeleton and Filament of Cellular Components Causing Gut Disruption in Growing–Finishing Pigs
Source: Animals (Basel). 2024 Aug 26;14(17):2476. doi: 10.3390/ani14172476 (PMC11394201; doi:10.3390/ani14172476)
Supplement: Supplementary file 1 [file animals-14-02476-s001.zip › Table S1.pdf]

Table S1. Genes differentially expressed in porcine jejunum (NT vs. HT).				
Gene symbol	Fold Change	P-value	Average of normalized data (log2)	
			NT	HS
LOC102166944	0.325	0.000	6.888	5.268
GPT2	0.472	0.000	4.658	3.575
HSPB6	2.191	0.000	6.224	7.356
PPP1R14A	2.194	0.000	5.389	6.522
CKM	3.128	0.000	3.706	5.352
SYNM	2.001	0.000	5.551	6.552
NEXN	2.026	0.049	3.100	4.119
TSPAN1	0.418	0.000	6.131	4.872
GSTA1	2.488	0.000	6.016	7.331
LOC106504562	2.012	0.000	5.772	6.781
GZMB	0.374	0.000	7.639	6.219
TAGLN	2.363	0.001	8.874	10.115
C4BPA	2.145	0.000	4.646	5.747
LOC110255503	0.366	0.000	4.194	2.744
DDC	0.407	0.000	5.162	3.866
CSRP1	2.047	0.000	6.886	7.919
TPM2	2.377	0.000	8.506	9.755
CCL4	0.467	0.022	4.123	3.026
ITGAE	0.431	0.000	4.612	3.398
PMP22	0.406	0.000	6.643	5.343
LOC110256379	2.293	0.000	3.210	4.407
MYLK	2.216	0.000	6.927	8.074
SH3BGR	2.097	0.000	3.716	4.784
PCP4	2.787	0.000	5.749	7.228
MX1	0.356	0.000	6.084	4.593
LOC110256649	20.023	0.019	0.536	4.860
C14H10orf99	0.452	0.000	5.340	4.194
PDLIM3	2.017	0.003	4.060	5.072
LOC106506288	17.331	0.000	0.000	4.115
DES	2.696	0.000	7.855	9.286
MYL9	2.248	0.000	7.815	8.984
PYGM	2.173	0.000	3.896	5.016
LOC110258673	0.399	0.001	4.207	2.882
CNN1	2.485	0.000	7.535	8.849
PDLIM7	2.001	0.000	5.052	6.052
GNLY	0.317	0.000	6.921	5.265
ACTG2	3.296	0.000	8.105	9.825
PLB1	0.468	0.000	5.122	4.028
FABP4	3.963	0.000	2.244	4.231
S100A2	2.226	0.000	4.019	5.173
TPM1	2.190	0.000	8.031	9.162

LOC110260659	0.128	0.001	6.132	3.160
MGP	2.374	0.000	7.204	8.451
