# Supplementary material for: Heat Stress Induces Alterations in Gene Expression of Actin Cytoskeleton and Filament of Cellular Components Causing Gut Disruption in Growing–Finishing Pigs
Source: Animals (Basel). 2024 Aug 26;14(17):2476. doi: 10.3390/ani14172476 (PMC11394201; doi:10.3390/ani14172476)
Supplement: Supplementary file 1 [file animals-14-02476-s001.zip › Table S2.pdf]

Table S2. Ingredients and nutrient composition in the experimental diets

**Ingredients (%)**	**Basal diet**
Corn	70.93
Wheat	5.00
Oil	1.71
SBM (44%)	19.38
Sugar Beet Pulp	0.00
DL-Methionine (98%)	0.03
L-Lysine (78.8%)	0.22
Threonine (99%)	0.03
Limestone	0.72
DCP	1.22
Salt	0.20
Choline	0.05
Mineral premix[1]	0.15
Vitamin premix[2]	0.15
$NaCO_3$	0.16
Phytase	0.05
Total	100.0
**Nutrients**	
Metabolizable Energy (kcal/kg)	3,300
Crude Protein (%)	15.20
Calcium (%)	0.62
Total Phosphorus (%)	0.30
Lysine (%)	0.90
Methionine + Cysteine (%)	0.51
Threonine (%)	0.56
Tryptophane (%)	0.17

[1]Supplied per kilogram diet: 62.1 mg Fe; 4.1 mg Cu; 59 mg Zn; 2.1 mg Mn; 0.19 mg Se; and 0.14 mg I.
[2]Supplied per kilogram diet: 1,400 IU vitamin A; 160 IU vitamin D3; 12 IU vitamin E; 0.51 mg vitamin K3; 1.1 mg thiamine; 2.7 riboflavin; 9 mg pantothenic acid; 35 mg niacin; 1.1 mg pyridoxine; 0.07 mg biotin; 0.4 mg folic acid; 10 µg vitamin B12; and 350 mg choline.
