# Supplementary figures and images for: Heat Stress Induces Alterations in Gene Expression of Actin Cytoskeleton and Filament of Cellular Components Causing Gut Disruption in Growing–Finishing Pigs
Source: Animals (Basel). 2024 Aug 26;14(17):2476. doi: 10.3390/ani14172476 (PMC11394201; doi:10.3390/ani14172476)

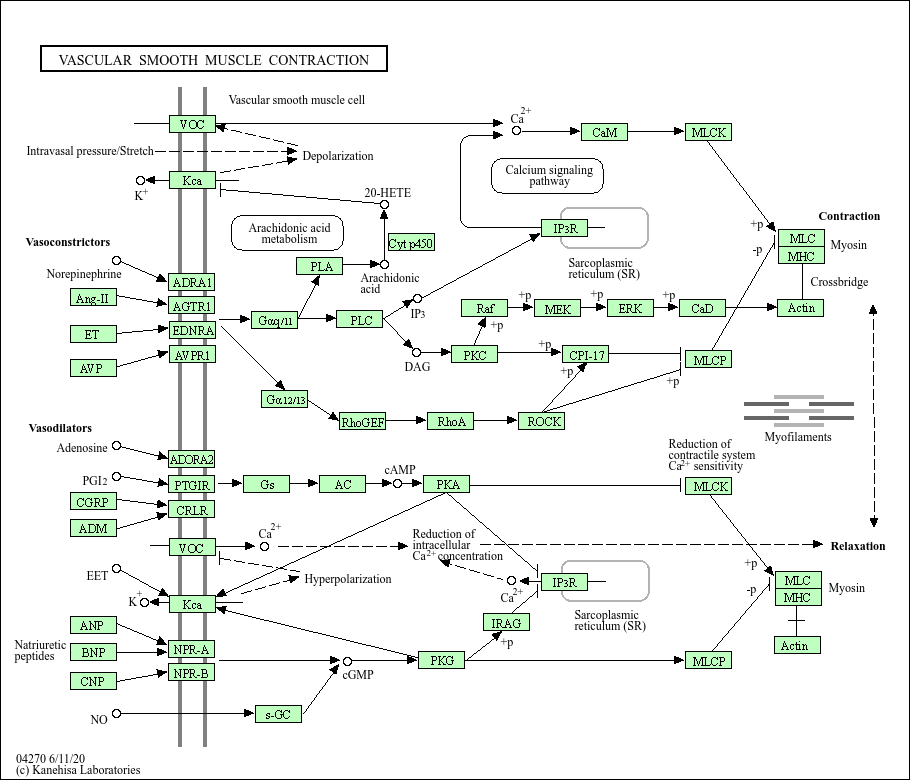

Supplement: Supplementary file 1 [file animals-14-02476-s001.zip › Figure S1.png]

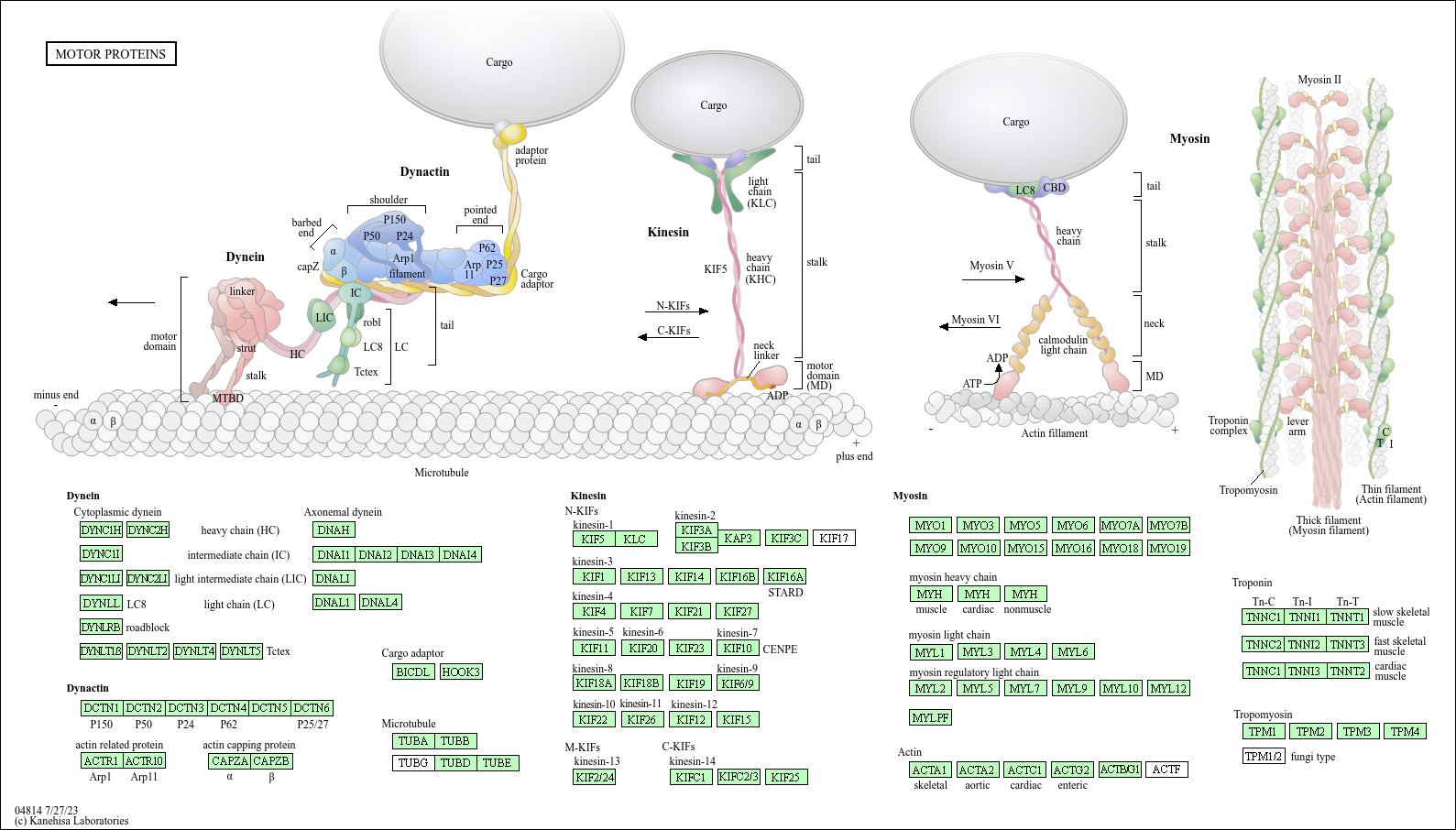

Supplement: Supplementary file 1 [file animals-14-02476-s001.zip › Figure S2.png]

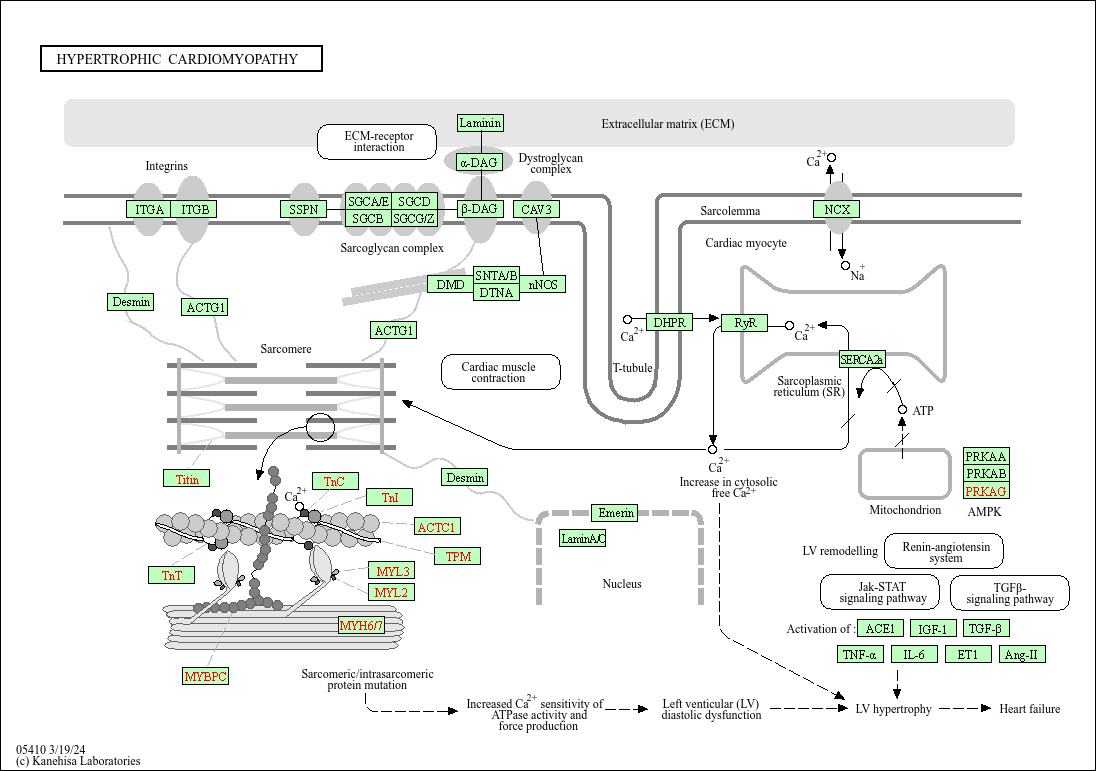

Supplement: Supplementary file 1 [file animals-14-02476-s001.zip › Figure S3.png]

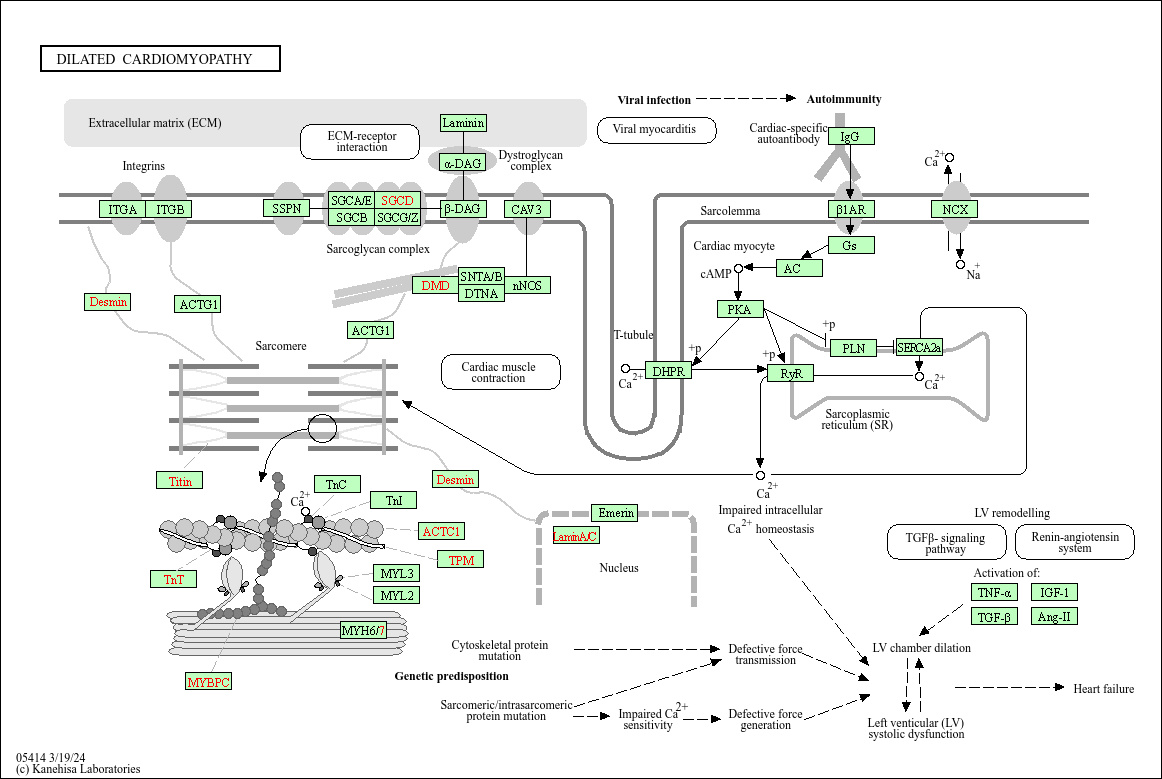

Supplement: Supplementary file 1 [file animals-14-02476-s001.zip › Figure S4.png]

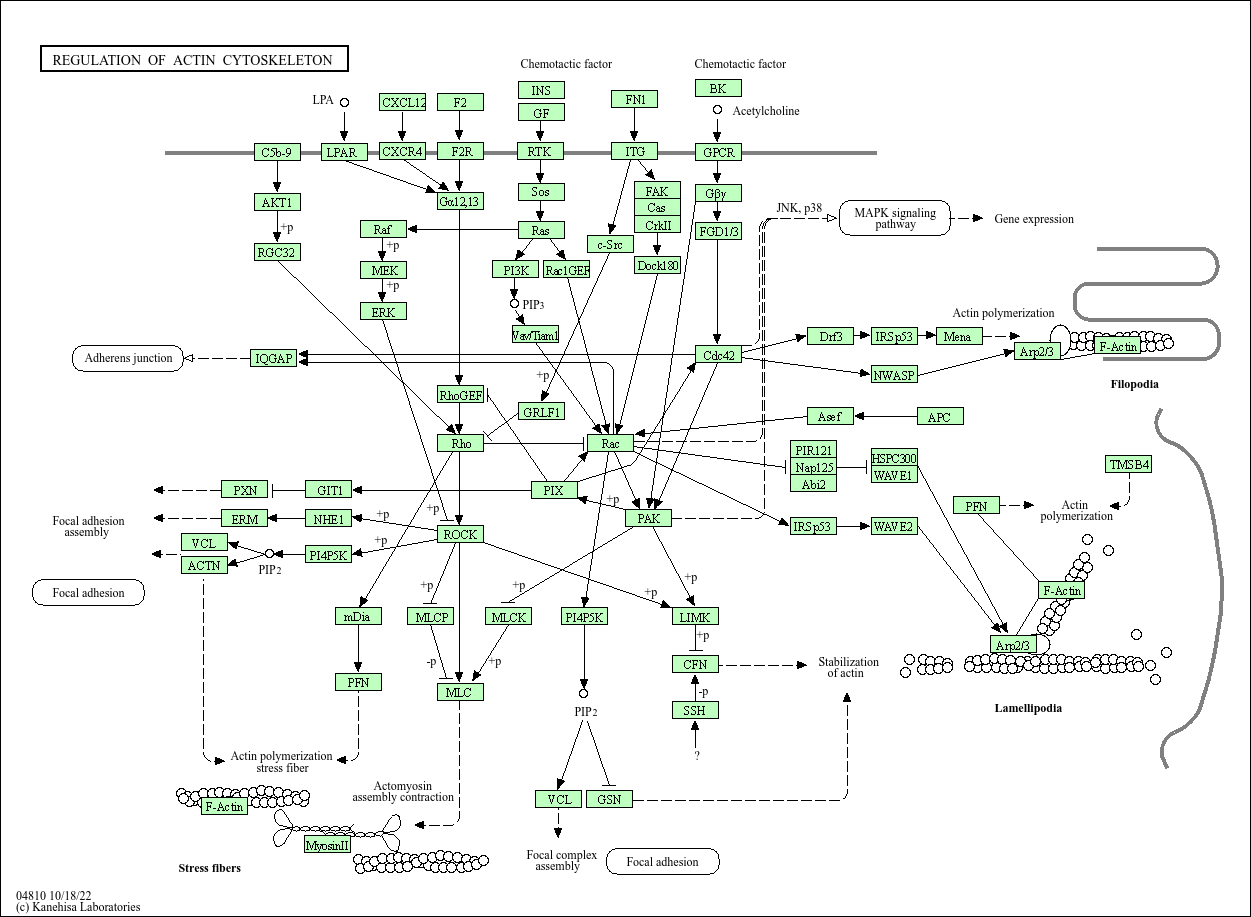

Supplement: Supplementary file 1 [file animals-14-02476-s001.zip › Figure S5.png]

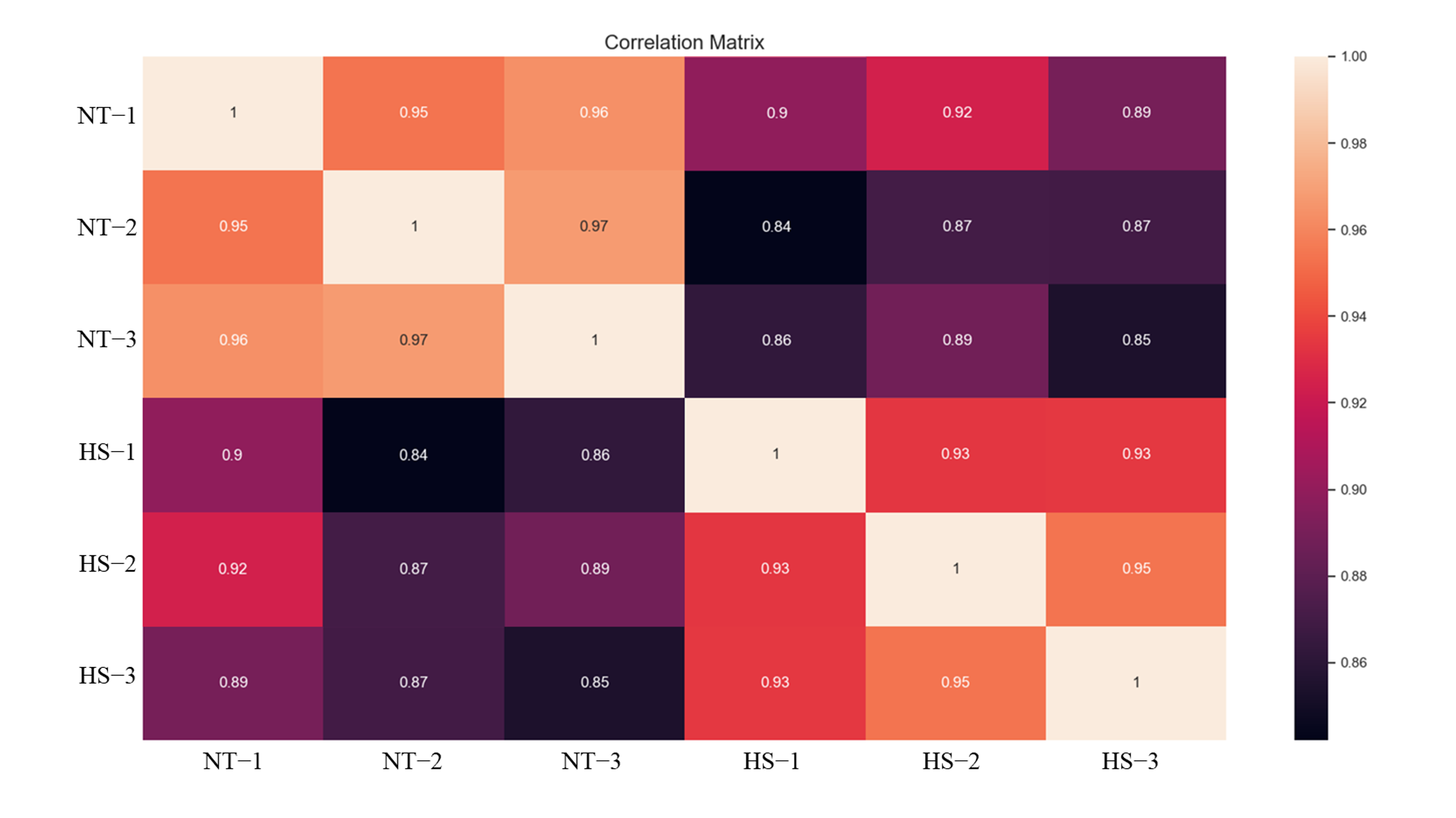

Supplement: Supplementary file 1 [file animals-14-02476-s001.zip › Figure S6.png]
